# Null results for the steal-framing effect on out-group aggression

**DOI:** 10.1038/s41598-021-04729-z

**Published:** 2022-01-13

**Authors:** Nobuhiro Mifune

**Affiliations:** School of Economics and Management, Kochi University of Technology, 2-22 Eikokuji, Kochi City, Kochi 780-8515 Japan

**Keywords:** Psychology, Human behaviour, Social evolution

## Abstract

Whether intergroup conflict is a necessary condition for the evolution of human prosociality has been a matter of debate. At the center of the debate is the coevolutionary model of parochial altruism—that human cooperation with in-group members has coevolved with aggression toward out-group members. Studies using the intergroup prisoner’s dilemma–maximizing difference game to test the model have repeatedly shown that people do not exhibit out-group aggression, possibly because of an inappropriate operationalization and framing of out-group aggression. The coevolutionary model predicts out-group aggression when the actor understands that it will lead to the in-group’s benefit. However, in the game, such an aspect of out-group aggression that benefits the in-group is typically not well communicated to participants. Thus, this study tested the hypothesis that out-group aggression in the game would be promoted by a framing that emphasizes that attacking out-group members enhances the in-group’s gain. Results of two laboratory experiments with 176 Japanese university students in total showed that such a framing did not promote out-group aggression and individuals invested more money to cooperate with in-group members only, avoiding the strategy of cooperating with in-group members to harm out-group members. These results do not support the coevolutionary model.

## Introduction

Intergroup conflict, including the extreme case of war, is ubiquitous in human social life^1–3^. To understand the nature of intergroup conflict, researchers from diverse disciplines, such as sociology, political science, economics, and other social sciences, have conducted studies to unravel its causes^4–6^. In social psychology, intergroup conflict has been attributed to intergroup bias^7,8^, which is the human tendency to behave altruistically or cooperatively toward members of the group to which they belong (i.e., in-group) and/or competitively or aggressively toward members of the group to which they do not belong (i.e., out-group). Various theories have been proposed as a psychological explanation of it^8–11^.

Recent experimental studies in social and evolutionary psychology have revealed that intergroup bias mainly stems from in-group cooperation, and out-group aggression rarely occurs in economic games^12–14^. However, based on an evolutionary perspective^15^, the absence of out-group aggression in previous studies may be due to the lack of a critical condition, such as a cue that attacking the out-group would increase the benefit to the in-group. Thus, the current study introduces such a cue to an experimental game paradigm and examines whether out-group aggression emerges.

### Coevolutionary model of intergroup bias

Intergroup bias is present even between arbitrarily created experimental groups (i.e., minimal groups)^16^. Intergroup bias in minimal groups—in which no conflict of interest, negative stereotypes, or face-to-face interactions within or between groups exist—has been consistently observed in student^17^ and non-student samples^18^ across various cultures^19,20^. Evolutionary and social psychologists have formed several hypotheses on the evolutionary origin of intergroup bias^21,22^.

One of the evolutionary hypotheses explaining intergroup bias is the coevolution model (CO model)^15,23,24^, which holds that humans simultaneously acquire in-group cooperation and out-group aggression. Choi and Bowles^15^ used an agent-based simulation and showed that agents who displayed both in-group and out-group aggression thrived, suggesting that in-group and out-group aggression co-evolved. The validity of the CO model has been examined using various datasets, ranging from ethnohistorical and archaeological^25^ to experimental^26,27^, although evidence for the model has been mixed^14,28–30^.

A feature that distinguishes the CO model from other evolutionary models of intergroup bias^22^ is the assertion of the existence of out-group aggression. Previous studies have typically employed the intergroup prisoners’ dilemma–maximizing difference game (IPD–MD)^12^ to test the CO model experimentally. In the IPD–MD, players are randomly divided into two groups. They receive a fixed amount of money from the experimenter. They decide how much to keep for themselves and to invest in the two pools: the within-group and the between-group pools. The total amount of money invested in the within-group pool by the members of the group is doubled and divided among in-group members equally. However, the total amount of money invested in the between-group pool is doubled; all in-group members receive an equal split *and* out-group members lose half the amount of the money (equal to the total invested amount before doubling). Specifically, any investment in the between-group pool includes harming out-group members, i.e., out-group aggression. If people are inclined to pursue self-interest, they would keep the entire amount of the initial endowment for themselves. If people are inclined only to cooperate with in-group members, they will invest their money in the within-group and/or between-group pools, without distinguishing between the two pools. Meanwhile, if people intend to cooperate with in-group members and harm out-group members, they would invest in the between-group pool that reduces the resource of out-group members.

The CO model predicts that individuals are inclined to invest more in the between-group pool than in the within-group pool in the IPD–MD^31^. Nevertheless, several studies have revealed that individuals, in general, invest more money in the within-group pool than in the between-group pool^12,32–35^. This pattern was also observed when researchers modified the game to encourage investment in the between-group pools^36–41^. Their findings offer conflicting evidence for the CO model: the empirical evidence does not suggest that individuals simultaneously acquire the tendency for in-group cooperation and out-group aggression.

### Out-group aggression in the IPD–MD

Behavioral evidence from studies using the IPD–MD does not support the CO model. Nonetheless, in-group and out-group aggression not evolving together would be a premature conclusion given the uncertainty of whether out-group aggression in the IPD–MD (i.e., investment in the between-group pool) corresponds to the operationalization of this concept in the CO model. Thus, the present study argues the vital importance of revisiting whether the IPD–MD offers contextual cues to elicit out-group aggression that is arguably acquired during evolution.

Human minds are thought to have domain-general and domain-specific features^42,43^, and evolutionarily acquired psychological mechanisms are often characterized as domain-specific^44^. For instance, the percentage of accuracy in performing logical reasoning tasks increases when the task is framed to detect norm violators^45,46^. That is, “cheater detection” is thought to be an adaptive reasoning system specific to the domain of social exchange^47^. Previous studies have identified various domain-specific psychological systems^47^, including theory of mind^48^, intuitive physics^49^, and folk biology^50^. Moreover, intergroup bias is also considered a domain-specific feature^51^.

Situational cues that imply a certain adaptive task reportedly activate a corresponding domain-specific mindset and behavior. For instance, disease priming, which induces pathogen infection threat assessment, leads to the preference for a symmetrical face, which indicates a high-functioning immune system^52^, and negative attitudes toward immigrants, who are associated with the possibility of unknown pathogens^53,54^. Cues of an important social exchange situation may also facilitate cooperation in a prisoner’s dilemma game^55,56^. If intergroup bias stems from an evolutionarily acquired domain-specific mindset^51^, then out-group aggression may be triggered by the cue of an adaptation task, such as intergroup conflict.

Choi and Bowles^15^ described out-group aggressions predicted by the CO model as in-group-benefitting behavior. One of the main causes of intergroup conflicts is the competition for resources, such as food, territory, and mating opportunities^57–59^. The in-group-benefitting aspect of out-group aggression is an important tenet of out-group aggression in the CO model. Several evolutionary models and simulations suggest that out-group aggression might evolve when individuals gain resources from an out-group^24,60^. Moreover, people display hostile intergroup behavior when out-group resources can be acquired^61^; people also tend to be sensitive to threats arising from hostile intergroup relationships^62,63^. This suggests that out-group aggression is favored when it benefits the in-group.

However, out-group aggression in IPD–MD seems to show a discrepancy with how it has been theoretically defined in the CO model. Specifically, previous studies using the IPD–MD have operationalized out-group aggression as simply reducing the payoff of out-group members: investment in the between-group pool is typically framed as merely about increasing and decreasing payoffs for in-group and out-group members, respectively^12^. Thus, the in-group-benefitting aspect of out-group aggression seems to be missing in the IPD–MD, and this might be why previous studies using the game have yielded inconsistent results with the CO model.

### The present research

The present research aimed to test the framing effect in an IPD–MD. Specifically, if out-group aggression in an IPD–MD (i.e., investment in the between-group pool) is framed such that it leads to financial gain for the in-group, then out-group aggression would emerge, which provides empirical support for the CO model.

Previous studies have revealed the framing effect on human decision making^64^, for example, the gain or loss framing on risky decision making^65^, attribute framing on evaluation^64^ and goal framing on cooperation^66^. Furthermore, the framing effect has been observed for evolutionary acquired domain-specific psychology^67^. This study examines whether out-group aggression would emerge when the contribution to the between-group pool is framed as having benefits to the in-group. Specifically, this study formulated two conditions that varied in the framing of the between-group pool: a control condition and a steal framing condition. In the former, the instructions on the two pools were identical to that of a basic IPD–MD. In the latter condition, the investment in the between-group pool was framed such that it led to moving money (like “stealing”) from an out-group to their own group.

The current research tested two hypotheses regarding the framing effect on out-group aggression. Böhm^31^ proposed the “weak” and “strong” hypotheses of out-group aggression based on the CO model: the amount invested in the between-group pool should be interpreted not only in terms of its absolute amount but also as a relative amount compared with the amount invested in the within-group pool, and “a larger contribution to the between-group pool relative to the within-group pool may be interpreted as negative attitude toward the out-group, an equal contribution to both pools as indifference regarding the out-group’s welfare, and a smaller contribution to the between-group pool as positive attitude toward the out-group”^13^ (p. 2). Based on the CO model, a negative attitude toward the out-group can be expected to lead to aggressive behavior in the steal-framing condition. Therefore, the present study set the “strong” hypothesis (hypothesis S) as follows:

#### Hypothesis S

In the steal condition, individuals would contribute more to the between-group pool than to the within-group pool.

Even if hypothesis S is not supported, as in previous studies^36,38,40,68^, steal framing may nonetheless lead to a less positive attitude. Therefore, the current study set the “weak” hypothesis (hypothesis W) as follows:

#### Hypothesis W

Individuals in the steal condition would invest more money in the between-group pool than those in the control condition.

The research aimed to further explore how the steal framing would influence out-group aggression. Framing effects in experimental economic games can be divided into effects of changes in preference and belief^69^. Preference tends to be stable regardless of the actions and reactions of an interacting partner. Meanwhile, belief refers to an expectation about others’ behavior. If the framing affected the investment in an IPD–MD, then whether the effect would be attributable to changes in preferences and/or beliefs merits investigation.

## Study 1

### Method

Study 1 was reviewed and approved by the ethical committee at Kochi University of Technology. In accordance with the Declaration of Helsinki, all participants provided written informed consent.

#### Participants

The study was advertised in a large student-participant pool at Kochi University of Technology, and 73 participants (34 women) voluntarily participated in the current study. Their mean age was 19.53 years (SD = 1.12).

#### Procedure

Upon arrival at the experimental lab, participants were given a participant ID and instructed not to share it with anyone. They were then escorted individually to an experimental cubicle, in which they completed the study on a computer without the physical presence of others. Once they were seated, they received instructions about the IPD–MD. Participants were first randomly divided into two groups, and each was given 600 JPY by the experimenter. Next, they had a one-shot interaction in which they each decided how much of the 600 JPY to keep for themselves and to invest in the within- and inter-group pools. The amount invested in the within-group pool was doubled by the experimenter and the total was distributed equally to all members of the participants’ own group.

Participants were randomly assigned to one of the two framing conditions (i.e., the control and steal conditions) and received different instructions on the between-group pool. In the control condition, they were informed that the total amount invested in the pool would be doubled and then distributed equally among in-group members regardless of their contribution. Moreover, they were told that out-group members would lose half the amount of money that in-group members received. The only difference between the control and the steal framing condition was whether the money invested in the between-group pool increased the in-group resource or decreased the out-group resource, as per the experimenter. For the control condition, the instructions mentioned that the experimenter increased the in-group’s money and decreased the out-group’s money. Specifically, in a game between Groups X and Y, each of which has five participants, if Participant A from Group X invests 100 JPY in the between-group pool, the experimenter doubles the amount of money and distributes it equally among the five participants. Therefore, all participants in Group X, including participant A, would receive 40 JPY each. Simultaneously, the experimenter deducts 20 JPY from each of the five participants in Group Y. Meanwhile, in the steal condition, the amount of money invested in the between-group pool moves from the out-group to the in-group. For example, if Participant A of Group X invests 100 JPY in the between-group pool, all members of Group X, including A, would receive 20 JPY each. They receive an additional 20 JPY that is taken from out-group members. In other words, the amount of money deducted from Group Y is given to Group X. Thus, the payoff structure of each individual was identical in both conditions. The word “steal” was not used in the instructions including the steal framing condition. The participants were told that all of them would decide simultaneously and that they could complete the game only once. It was also emphasized that the rules of the game were the same for out-group members. After reading the instructions about the IPD–MD, the participants input their decisions on a computer screen.

The participants then completed a post-experiment questionnaire. They first answered the comprehension and manipulation check questions. The tool contained three comprehension check questions: (C1) “Whose money does the investment in the within-group pool increase?” (C2) “Whose money does the investment in the between-group pool increase?” and (C3) “Whose money does the investment in the between-group pool increase?” They selected one of the following options: “increase (decrease) the money of my group as a whole,” “increase (decrease) the money of others’ group as a whole,” “increase (decrease) the money of both groups,” “increase (decrease) the money of neither group,” or “don’t know.” For the manipulation check, they were asked the following question: “Who received the money subtracted from the members of the other group owing to the investment in the between-group pool?” They answered this with one of the following options: “the money went to my group,” “the experimenter collected the money,” or “I don’t know.”

After the comprehension and manipulation checks, the participants inferred how other members of their own group and the other group had completed the game. Specifically, they were asked how much other members of their ingroup and outgroup had kept and invested in the within- and between-group pools, in units of 50 JPY. For exploratory analyses, the study administered items measuring identification with the in-group and out-group and social dominance orientation (see S4 for more details). Finally, the participants provided demographic information (e.g., sex and age), received rewards according to the results of the game. The entire experiment took approximately 50 min.

### Results

#### Comprehension and manipulation checks

The correct answer rates for C1, C2, and C3 were 97.3%, 91.8%, and 90.4%, respectively. We found no significant differences in the correct response rate between conditions (Fisher’s exact tests: all *p*s > 0.05). The participants’ understanding of the rules of the game was satisfactory. The correct response rate for the manipulation check was 94.5%. In the control condition, 89.2% of the participants answered correctly, and 10.8% answered incorrectly or chose “I don’t know.” All participants in the steal condition answered correctly. The correct response rate between the two conditions showed no significant difference (Fisher’s exact test, *p* = 0.115). As most of the participants correctly answered the comprehension and manipulation check questions, we did not conduct data exclusion. Data exclusion based on these questions did not change results in a meaningful manner (see S1).

#### Behavioral data analysis

**Figure 1:**
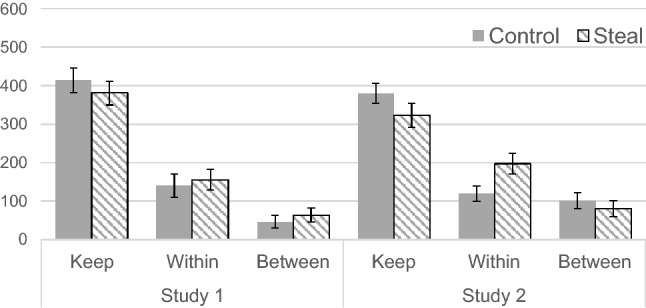
Mean keep or invest amounts for each condition (error bars represent standard errors).

On average, participants kept 397.26 JPY (SD = 191.10), invested 147.95 JPY (SD = 171.28) in the within-group pool, and invested 54.79 JPY (SD = 103.47) in the between-group pool. The means and standard deviations of their investments under different conditions are shown in Fig. 1. Following Halevy et al.^12^, the study conducted a 2 (framing: control vs. steal) × 2 (pool: within- vs. between-group) mixed ANOVA. The results showed a significant main effect of pool, *F*(1, 71) = 14.33, *p* < 0.001, *η*_p_^2^ = 0.17. The main effects of framing (*F*(1, 71) = 0.54, *p* = 0.465, *η*_p_^2^ = 0.01) and the interaction effect (*F*(1, 71) = 0.004, *p* = 0.953, *η*_p_^2^ < 0.001) were insignificant.

#### Hypothesis testing

The mean amount of investment in the between-group pool was 45.94 JPY (SD = 100.95) in the control condition and 63.89 JPY (SD = 106.64) in the steal condition, with no significant difference between them (*t*(71) = 0.74, *p* = 0.46, d = 0.17). In the steal condition, the participants invested a significantly lower amount in the between-group pool than the within-group pool (M = 155.56, SD = 159.81, *t*(35) = 2.75, *p* = 0.009, d = 0.68). Therefore, hypotheses S and W were not supported. Using G*Power 3.1^69^, sensitivity analysis of a two tailed t-test (power = 0.8) for hypothesis S revealed that the detectable effect size was 0.665 and that for hypothesis W was 0.48. This suggests that the observed effect sizes, especially for hypothesis S, were not big enough for the current study to detect.

#### Inference about others’ behavior

**Figure 2:**
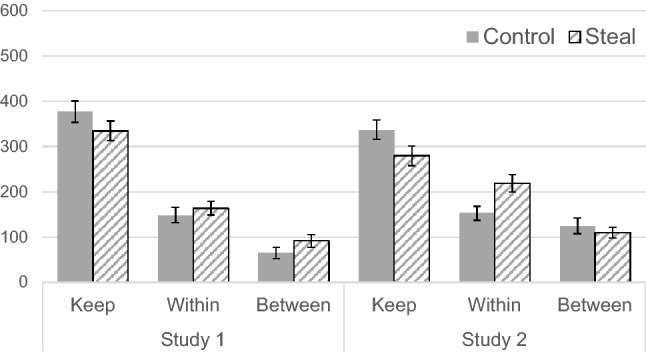
Mean estimated keep or invest amounts by other in-group members in each condition (error bars represent standard errors).

**Figure 3:**
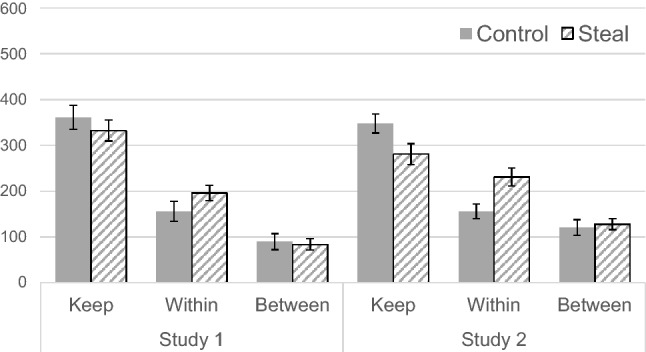
Mean estimated keep or invest amounts by out-group members in each condition (error bars represent standard errors).

Although the effect of the framing manipulation was not significant, we examined the participants’ inferences about other in-group and out-group members’ investment decisions (see Figs. 2, 3, and Fig. S3). The A 2 (framing: control vs. steal) × 2 (pool: within- vs. between-group) mixed ANOVA on the estimated amount of money invested by in-group members revealed that the main effect of the pool was significant, *F*(1, 71) = 29.26, *p* < 0.001, *η*_p_^2^ = . 29. The main effect of framing (*F*(1, 71) = 1.90, *p* = 0.173, *η*_p_^2^ = 0.03) and the interaction effect (*F*(1, 71) = 0.16, *p* = 0.690, *η*_p_^2^ = 0.002) were not significant. Thus, the steal framing did not alter the expectation about how other in-group members would complete the game.

Further, the A 2 (framing) × 2 (pool) mixed ANOVA on the estimated amount of money invested by out-group members revealed a significant main effect of pool, *F*(1, 71) = 23.15, *p* < 0.001, *η*_p_^2^ = 0.25. The main effect of framing (*F*(1, 71) = 1.09, *p* = 0.299, *η*_p_^2^ = 0.02) and the interaction effect (*F*(1, 71) = 1.55, *p* = 0.217, *η*_p_^2^ = 0.02) were not significant. This indicates that the framing did not alter the behavioral expectation of out-group members.

### Discussion

Both hypotheses were not supported in Study 1: results showed that steal framing did not increase out-group aggression in IPD–MD. Thus, even when out-group aggression was framed as being beneficial for the in-group, the CO model was not empirically supported.

Study 1 had fewer than 40 participants in each condition, smaller than the number of participants recruited in the majority of studies that used the IPD–MD^31,38,68^; except for Halevy et al.^40^. As per the sensitivity analysis, the hypotheses were not supported, possibly due to the small sample size. Thus, we conducted Study 2 with more participants and re-tested the hypotheses.

## Study 2

### Method

Study 2 was reviewed and approved by the ethical committee at the Kochi University of Technology. In accordance with the Declaration of Helsinki, all participants provided written informed consent.

#### Participants and procedure

Study 2 intended to recruit 50 participants for each condition. A total of 107 Japanese undergraduate students signed up for the study (53 females, 51 males, and 3 neither or respondent refusals). Their mean age was 18.87 years (SD = 1.18). Participants were recruited from a large participant pool at Kochi University of Technology with an emphasis on monetary incentives. The procedure and materials were identical to those in study 1.

### Results

#### Comprehension and manipulation checks

The correct answer rates for the three comprehension check questions were 96.1%, 91.3%, and 94.2%. The two framing conditions showed no significant differences (Fisher’s exact test: all *p*s > 0.05). The correct response rate for the manipulation check item was 91.3%. In the control condition, 83.3% of the participants answered it correctly, and 16.7% answered it incorrectly or chose “I don’t know.” All (100%) the participants answered correctly in the steal condition, and the difference was significant (Fisher’s exact test: *p* = 0.003). The exclusion of participants in the control condition who failed to answer the questions correctly did not change the results of subsequent analyses in a meaningful way. Thus, results without data exclusion are reported here (see S2 for analyses with data exclusion).

#### Behavioral data analysis

The participants, on average, kept 352.43 JPY (SD = 202.84) for themselves and contributed 156.31 JPY (SD = 170.31) and 91.26 JPY (SD = 150.72) to the within- and between-group pools, respectively (see Fig. 1 for the descriptive statistics by condition). A 2 (framing: control vs. steal) × 2 (pool: within- vs. between-group) mixed ANOVA on the amount of investment revealed a significant main effect of pool (*F*(1, 101) = 7.72, *p* = 0.007, *η*_p_^2^ = 0.07) and a significant interaction effect (*F*(1, 101) = 4.06, *p* = 0.047, *η*_p_^2^ = 0.04). In contrast, the main effect of framing was not significant, *F*(1, 101) = 2.06, *p* = 0.154, *η*_p_^2^ = 0.02. Post hoc comparisons using the Holm method revealed that the amount of investment in the within-group pool was higher in the steal condition than that in the control condition (*p* = 0.014), but the difference in investment in the between-group pool between the two conditions was not significant (*p* = 0.518). In addition, the amount of investment between the within- and between-group pools in the control condition did not significantly differ (*p* = 0.581). Meanwhile, the difference in the steal condition was significant (*p* = 0.001).

#### Hypothesis testing

The mean amount of investment in the between-group pool was 100.93 JPY (SD = 156.76) in the control condition and 80.61 JPY (SD = 144.63) in the steal condition, with no significant difference between them (*t*(101) = 0.68, *p* = 0.497, d = 0.13). In the steal condition, the amount invested in the between-group pool was significantly lower than that in the within-group pool (M = 196.94, SD = 187.47, *t*(48) = 3.18, *p* = 0.003, d = 0.70). Therefore, hypotheses S and W were not supported. Sensitivity analysis of a two tailed t-test (power = 0.8) for hypothesis S revealed that the detectable effect size was 0.558 and that for hypothesis W was 0.408. This suggests that the observed effect sizes, especially for hypothesis S, were not big enough for the current study to detect.

#### Inference about others’ behavior

We examined the participants’ inferences about other in-group and out-group members’ investment decisions (see Figs. 2, 3, and Fig. S4). The A 2 (framing) × 2 (pool) mixed ANOVA on the inferred investment of in-group members revealed that the main effect of pool (*F*(1, 101) = 15.32, *p* < 0.001, *η*_p_^2^ = 0.13) and the interaction effect (*F*(1, 101) = 5.36, *p* = 0.023, *η*_p_^2^ = 0.05) were significant. Meanwhile, the main effect of framing was not significant, *F*(1, 101) = 2.8, *p* = 0.097, *η*_p_^2^ = 0.03. Post hoc pairwise comparisons with the Holm method revealed that the level of inferred investment in the between-group pool was not different between the two framing conditions (*p* = 0.52). In contrast, the inferred investment in the within-group pool in the steal condition was significantly higher compared with that of the control condition (*p* = 0.005). These results showed that although steal framing increased the inference of cooperation among in-group members, it did not increase the inference that other in-group members would attack out-group members.

The same ANOVA on the inferred investment out-group members revealed that the main effect of framing (*F*(1, 101) = 7.19, *p* = 0.009, *η*_p_^2^ = 0.07) and pool (*F*(1, 101) = 15.35, *p* < 0.001, *η*_p_^2^ = 0.13) were significant. The interaction effect was not significant (*F*(1, 101) = 3.7, *p* = 0.057, *η*_p_^2^ = 0.04). Thus, steal framing generated an inference that out-group members would no longer pursue self-interest, but this did not mean that they would invest more in out-group aggression.

### Discussion

Study 2 showed the same patterns as those in Study 1. The amount of investment in the between-group pool in IPD–MD did not increase with steal framing and exceeded the amount invested in the within-group pool. These results were inconsistent with the CO model.

## General discussion

We tested the framing effect on out-group aggression in the IPD–MD. In both studies 1 and 2, steal framing did not promote aggression toward out-group members, and individuals preferred in-group cooperation without harming out-group members regardless of the game framing. Thus, these studies did not provide support for the hypotheses that were based on the CO model.

Previous studies using experimental economic games, such as the public goods game, have shown that framing can change behavior^70–73^. In the IPD–MD, group framing promotes aggression toward out-group members^68^. Given that framing manipulations can affect behavior in the IPD–MD, the findings from the two studies can be interpreted as suggesting that a cue of out-group deprivation (i.e., stealing money from them) does not promote out-group aggression.

In the current study, regardless of the framing condition, the amount of investment in the between-group pool was not higher than that in the within-group pool, consistent with previous findings^14,68^. These findings support another evolutionary model of intergroup bias, namely, bounded generalized reciprocity (BGR)^17,22,74^. The BGR model holds that in-group bias stems from cooperation with in-group members rather than aggression toward out-group members^20,69,75^. The BGR model assumes that people have an intuitive belief that indirect reciprocity exists within the group, and that acting cooperatively toward in-group members is of vital importance to establishing a good reputation. However, individuals do not suppose that out-group members are part of the system of indirect reciprocity, and consequently, they are not motivated to display altruistic behavior to earn a reputation not only with respect to out-group members but also strangers. In other words, the BGR model assumes that humans do not act altruistically toward out-group members, not that they proactively attack them. Although the current study did not aim to validate the BGR model, the results are consistent with the model.

Out-group aggression can emerge when it can defend an ingroup^76,77^. For example, Böhm et al.^36^ showed that, although not the framing manipulation, the amount of investment in the between-group pool increases when it can prevent attacks from the out-group. Additionally, the framing instruction used in Weisel and Zultan^68^, which supports the “weak hypothesis” in IPD–MD, seems to have an in-group defense-like phrase. Further research could investigate whether out-group aggression can occur by setting framing characterized by a defensive function.

The CO model has been criticized not only for its theoretical assumptions^30,78^ but also for the conflicting empirical findings^77,79,80^. These do not necessarily mean, however, that humans have not evolutionarily acquired any aggressive tendency toward out-group members. There may be several types of out-group aggression^76^ that are possibly suited for the CO model, for example, preemptive strikes^81^ and proactive or reactive aggression^82^. A theoretical and empirical examination of the types of aggression directed toward out-group members is necessary.

There are other two limitations. First, framing manipulation was used to emphasize that out-group aggression increases the benefit of the in-group, but it is not clear whether the framing changed the participants’ perception as intended. That said, participants in the steal framing condition might see the investment in the between-group pool as a means to decrease the benefit of the out-group rather than increase the benefit of the in-group. In that case, they might refrain from investing money in the between-group pool because it would be socially undesirable. Second, as per the sensitivity analysis, the effect may not have been detected because the sample size was small. Compared to the effect sizes obtained in previous meta-analyses on the gain–loss framing^83,84^, the effect size of *d* = 0.17 obtained in study 1 was relatively small. Additionally, the effect size of *d* = 0.13 obtained in study 2 was in the opposite direction of study 1, and the combined effect size of study 1 and 2 was close to zero (d = 0.04, see S3). Therefore, the effect size of steal framing, even if it existed, would most likely be small.

## Supplementary Information

Supplementary Information.

## Data Availability

The data associated with this research are temporarily available at [https://osf.io/8sgeh/].

## References

[1] Brown DE (1991). Human Universals.

[2] Gat A (2006). War in Human Civilization.

[3] Sumner WG (1906). Folkways.

[4] Kestnbaum M (2009). Sociology of war and the military. Annu. Rev. Sociol..

[5] Schelling TC (1980). Strategy of Conflict.

[6] Waltz KN (1959). Man, the State, and War: A Theoretical Analysis.

[7] Brewer MB (1979). In-group bias in the minimal intergroup situation: cognitive-motivational analysis. Psychol. Bull..

[8] Tajfel H, Turner JC, Austin WG, Worchel S (1979). Integrative theory of intergroup conflict. The Social Psychology of Intergroup Relations.

[9] Sidanius J, Pratto F (1999). Social Dominance: Intergroup Theory of Social Hierarchy and Oppression.

[10] Turner JC (1985). Social categorization and self-concept: A social cognitive theory of group behavior. Adv. Group Process..

[11] Böhm R, Rusch H, Baron J (2020). Psychology of intergroup conflict: A review of theories and measures. J. Econ. Behav. Organ..

[12] Halevy N, Bornstein G, Sagiv L (2008). “In-group love” and “out-group hate” as motives for individual participation in intergroup conflict: A new game paradigm. Psychol. Sci..

[13] Yamagishi T, Mifune N (2009). Social exchange and solidarity: in-group love or out-group hate?. Evol. Hum. Behav..

[14] Yamagishi T, Mifune N (2016). Parochial altruism: Does it explain modern human group psychology?. Curr. Opin. Psychol..

[15] Choi JK, Bowles S (2007). Coevolution of parochial altruism and war. Science.

[16] Tajfel H, Billig MG, Bundy RP, Flament C (1971). Social categorization and intergroup behavior. Eur. J. Soc. Psychol..

[17] Yamagishi T, Mifune N (2008). Does shared group membership promote altruism? Fear, greed, and reputation. Ration. Soc..

[18] Mifune N, Yamagishi T (2015). Correlation between ingroup favoritism and fear of negative evaluation. Jpn. J. Soc. Psychol..

[19] Romano A, Balliet D, Yamagishi T, Liu JH (2017). Parochial Trust and Cooperation across 17 Societies. Proc. Natl. Acad. Sci. U. S. A..

[20] Yamagishi T, Mifune N, Liu JH, Pauling J (2008). Exchanges of group-based favors: Ingroup bias in the prisoner’s dilemma game with minimal groups in Japan and New Zealand. Asian J. Soc. Psychol..

[21] Van Vugt M, De Cremer D, Janssen DP (2007). Gender differences in cooperation and competition: The male-warrior hypothesis. Psychol. Sci..

[22] Yamagishi T, Jin N, Kiyonari T (1999). Bounded generalized reciprocity: Ingroup boasting and ingroup favoritism. Adv. Group Process..

[23] García J, van den Bergh JCJM (2011). Evolution of parochial altruism by multilevel selection. Evol. Hum. Behav..

[24] Lehmann L, Feldman MW (2008). War and evolution of belligerence and bravery. Proc. R. Soc. B.

[25] Bowles S (2009). Did warfare among ancestral hunter-gatherers affects the evolution of human social behavior?. Science.

[26] Abbink K, Brandts J, Herrmann B, Orzen H (2012). Parochial altruism in intergroup conflicts. Econ. Lett..

[27] Bernhard H, Fischbacher U, Fehr E (2006). Parochial altruism in humans. Nature.

[28] Ferguson BF, Fry DP (2013). Pinker’s list: Exaggerating prehistoric war mortality. War, Peace, and Human Nature: The Convergence of Evolutionary and Cultural Views.

[29] Ferguson BF, Fry DP (2013). The prehistory of war and peace in Europe and the East. War, Peace, and Human Nature: The Convergence of Evolutionary and Cultural Views.

[30] Rusch H (2014). The evolutionary interplay of intergroup conflict and altruism in humans: A review of parochial altruism theory and prospects for its extension. Proc. R. Soc. B..

[31] Böhm R (2016). Intuitive participation in aggressive intergroup conflict: Evidence of weak versus strong parochial altruism. Front. Psychol..

[32] De Dreu CKW (2010). Social value orientation moderates ingroup love, but does not outgroup hate in competitive intergroup conflict. Group Process. Intergroup Relat..

[33] Halevy N, Weisel O, Bornstein G (2012). “In-group love” and “out-group hate” in repeated interaction between groups. J. Behav. Decis. Mak..

[34] Salvati M, Giacomantonio M, Ten Velden F (2020). Dispositional mindfulness moderates the association between social value orientation, in-group love, and out-group hates. Curr. Psychol..

[35] Thielmann I, Böhm R (2016). Who does (not) participate in intergroup conflict?. Soc. Psychol. Personal. Sci..

[36] Böhm R, Rusch H, Gürerk Ö (2016). What makes people go to war? Defensive intentions motivate retaliatory and preemptive intergroup aggression. Evol. Hum. Behav..

[37] Dang J, Ekim ZE, Ohlsson S, Schiöth HB (2020). Is there a prejudice from thin air? Replicating the effect of emotion on automatic intergroup attitudes. BMC Psychol..

[38] De Dreu CK, Dussel DB, Ten Velden FS (2015). In intergroup conflict, self-sacrifice is stronger among pro-social individuals, and parochial altruism emerges especially among cognitively taxed individuals. Front. Psychol..

[39] De Dreu CKW (2010). The neuropeptide oxytocin regulates parochial altruism during intergroup conflicts among humans. Science.

[40] Halevy N, Chou EY, Cohen TR, Bornstein G (2010). Relative deprivation and intergroup competition. Group Process. Intergroup Relat..

[41] Ten Velden FS, Daughters K, De Dreu CKW (2017). Oxytocin promotes intuitive rather than deliberate cooperation with the in-group. Horm. Behav..

[42] Bjorklund DF, Pellegrini AD (2002). The Origins of Human Nature: Evolutionary Developmental Psychology.

[43] Confer JC (2010). Evolutionary psychology: Controversies, questions, prospects, and limitations. Am. Psychol..

[44] Cosmides L, Tooby J, Hirschfeld L, Gelman S (1994). Origins of domain specificity: Evolution of functional organization. Mapping the Mind: Domain Specificity in Cognition and Culture.

[45] Cosmides L (1989). The logic of social exchange: Has natural selection shaped how humans reason? Studies using the Wason Selection Task. Cognition.

[46] Cosmides L, Barrett HC, Tooby J (2010). Adaptive specializations, social exchange, and the evolution of human intelligence. Proc. Natl. Acad. Sci. U. S. A..

[47] Cosmides L, Tooby J (2013). Evolutionary psychology: New perspectives on cognition and motivation. Annu. Rev. Psychol..

[48] Baron-Cohen S, Wheelwright S, Spong A, Scahill V, Lawson J (2001). Studies of theory of mind: Intuitive physics and intuitive psychology are independent. J. Dev. Phys. Disabil..

[49] Spelke ES (1990). Principles of object perception. Cogn. Sci..

[50] Mahon BZ, Caramazza A (2009). Concepts and categories: Cognitive neuropsychological perspective. Annu. Rev. Psychol..

[51] Geary DC (1998). Male and Female: Evolution of Human Sex Differences.

[52] Young SG, Sacco DF, Hugenberg K (2011). Vulnerability to disease is associated with a domain-specific preference for symmetrical faces relative to symmetrical non-face stimuli. Eur. J. Soc. Psychol..

[53] Faulkner J, Schaller M, Park JH, Duncan LA (2004). Evolved disease-avoidance mechanisms and contemporary xenophobic attitudes. Group Process. Intergroup Relat..

[54] Huang JY, Sedlovskaya A, Ackerman JM, Bargh JA (2011). Immunizing against prejudice: Effects of disease protection on attitudes toward out-groups. Psychol. Sci..

[55] Kiyonari T, Tanida S, Yamagishi T (2000). Social exchange and reciprocity: Confusion or a heuristic?. Evol. Hum. Behav..

[56] Yamagishi T, Terai S, Kiyonari T, Mifune N, Kanazawa S (2007). Social exchange heuristic: Managing errors in social exchange. Ration. Soc..

[57] Ember CR, Ember M (1992). Resource unpredictability, mistrust, and war: A cross-cultural study. J. Conflict Resolut..

[58] Gat A (2009). So why do people fight? Evolutionary theory and the causes of war. Eur. J. Int. Relat..

[59] Kohler T, Turner KK (2006). Raiding women in the pre-Hispanic northern Pueblo southwest? A pilot examination. Curr. Anthropol..

[60] Rusch H (2014). The two sides of warfare: An extended model of altruistic behavior in ancestral human intergroup conflict. Hum. Nat..

[61] Sherif M, Harvey OJ, White BJ, Hood WR, Sherif CW (1961). Intergroup Conflict and Cooperation: The Robbers Cave Experiment.

[62] Stephan WG, Stephan CW (1996). Predicting prejudice. Int. J. Intercult. Relat..

[63] Stephan WG, Ybarra O, Rios K, Nelson T (2016). Intergroup threat theory. Handbook of Prejudice, Stereotyping, and Discrimination.

[64] Levin IP, Schneider SL, Gaeth GJ (1998). All frames are not created equal: A typology and critical analysis of framing effects. Organ. Behav. Hum. Decis. Process..

[65] Tversky A, Kahneman D (1981). The framing of decisions and the psychology of choice. Science.

[66] Brewer MB, Kramer RM (1986). Choice behavior in social dilemmas: Effects of social identity, group size, and decision framing. J. Pers. Soc. Psychol..

[67] Saad G, Gill T (2014). The framing effect when evaluating prospective mates: An adaptationist perspective. Evol. Hum. Behav..

[68] Weisel O, Zultan R (2021). Perceptions of conflict: Parochial cooperation and outgroup spite revisited. Organ. Behav. Hum. Decis. Process..

[69] Faul F, Erdfelder E, Buchner A, Lang A-G (2009). Statistical power analyses using G*Power 3.1: Tests for correlation and regression analyses. Behav. Res. Methods.

[70] Columbus S, Münich J, Gerpott FH (2020). Playing a different game: Situation perception mediates the framing effects on cooperative behavior. J. Exp. Soc. Psychol..

[71] Andreoni J (1995). Warm-glow versus cold-prickle: Effects of positive and negative framing on cooperation in experiments. Q. J. Econ..

[72] Liberman V, Samuels SM, Ross L (2004). The name of the game: Predictive power of reputations versus situational labels in determining prisoner’s dilemma game moves. Pers. Soc. Psychol. Bull..

[73] List JA (2007). On the interpretation of giving in dictator games. J. Polit. Econ..

[74] Jin N, Yamagishi T (1997). Group heuristics in social dilemma. Jpn. J. Soc. Psychol..

[75] Balliet D, Wu J, De Dreu CKW (2014). Ingroup favoritism in cooperation: A meta-analysis. Psychol. Bull..

[76] De Dreu CK (2016). In-group defence, out-group aggression, and coordination failures in intergroup conflict. Proc. Natl. Acad. Sci. U. S. A..

[77] Mifune N, Simunovic D, Yamagishi T (2017). Intergroup biases in fear-induced aggression. Front. Psychol..

[78] Dyble M (2021). The evolution of altruism through war is highly sensitive to the population structure and to civilian and fighter mortality. Proc. Natl. Acad. Sci. U. S. A..

[79] Aaldering H, Böhm R (2020). Parochial versus universal cooperation: Introducing a novel economic game of within- and between-group interactions. Soc. Psychol. Personal. Sci..

[80] Aaldering H, Ten Velden FS, van Kleef GA, De Dreu CKW (2018). Parochial cooperation in nested intergroup dilemmas is reduced when it harms out-groups. J. Pers. Soc. Psychol..

[81] Simunovic D, Mifune N, Yamagishi T (2013). Preemptive strike: An experimental study of fear-based aggression. J. Exp. Soc. Psychol..

[82] Wrangham RW (2018). There are two types of aggression in human evolution. Proc. Natl. Acad. Sci. U. S. A..

[83] Kühberger A (1998). The influence of framing on risky decisions: A meta-analysis. Organ. Behav. Hum. Decis. Process..

[84] McDonald K, Graves R, Yin S, Weese T, Sinnott-Armstrong W (2021). Valence framing effects on moral judgments: A meta-analysis. Cognition.

