# Supplementary material for: Null results for the steal-framing effect on out-group aggression
Source: Sci Rep. 2022 Jan 13;12:686. doi: 10.1038/s41598-021-04729-z (PMC8758705; doi:10.1038/s41598-021-04729-z)
Supplement: Supplementary file 1 — Supplementary Information. [file 41598_2021_4729_MOESM1_ESM.docx]

Supplementary Materials

S1. Re-analysis with Data Exclusion for Study 1

On average, participants kept 392.86 JPY (SD = 191.73) and invested 151.43 JPY (SD = 172.99) in the within-group pool and 55.71 JPY (SD = 105.14) in the between-group pool. A 2 (framing: control vs. steal) × 2 (pool: within- vs. between-group) mixed ANOVA revealed a significant main effect of pool, *F*(1, 68) = 14.01, *p* < .001, *η*_p_^2^ = .17. The main effect of framing (*F*(1, 68) = 0.30, *p* = .584, *η*_p_^2^ = .004) and interaction effect (*F*(1, 68) = 0.03, *p* = .871, *η*_p_^2^ <.001) were not significant.

The mean amount of investment in the between-group pool was 47.06 JPY (SD = 104.40) in the control condition and 63.89 JPY (SD = 106.64) in the steal condition, with no significant difference between them (*t*(68) = 0.67, *p* = .507, d = 0.16). In the steal condition, the amount invested in the between-group pool was significantly lower than that in the within-group pool (M = 155.56, SD = 159.81, *t*(35) = 2.75, *p* = .009, d = 0.68). Therefore, hypotheses S and W were not supported.

S2. Re-analysis with Data Exclusion for Study 2

Participants, on average, kept 347.37 JPY (SD = 202.56) for themselves and contributed to the within- and between-group pools with 156.32 JPY (SD = 168.42) and 96.32 JPY (SD = 155.61), respectively (see Figure 2 for descriptive statistics by conditions). A 2 (framing: control vs. steal) × 2 (pool: within- vs. between-group) mixed ANOVA on the amount of investment revealed a significant main effect of pool (*F*(1, 93) = 5.23, *p* = .024, *η*_p_^2^ = .05) and a significant interaction effect (*F*(1, 93) = 5.23, *p* = .024, *η*_p_^2^ = .05). In contrast, the main effect of framing was not significant, *F*(1, 93) = 1.54, *p* = .218, *η*_p_^2^ = .02. Post hoc comparisons using the Holm method revealed that the amount of investment in the within-group pool was higher in the steal condition than in the control condition (*p* = .011). Meanwhile, the difference in investment in the between-group pool between the two conditions was not significant (*p* = .324). The amount of investment between within-group and between-group pools in the control condition did not significantly differ (*p* = 1.0), whereas the difference in the steal condition was significant (*p* = .001).

The mean amount of investment in the between-group pool was 113.04 JPY (SD = 166.48) in the control condition and 80.61 JPY (SD = 144.63) in the steal condition, with no significant difference between them (*t*(93) = 1.02, *p* = .313, d = 0.21). In the steal condition, the amount invested in the between-group pool was significantly lower than the amount invested in the within-group pool (M = 196.94, SD = 187.47, *t*(94) = 2.31, *p* = .023, d = 0.37). Therefore, hypotheses S and W were not supported.

S3. Means and Standard Deviations of Inference about others’ behavior in Study 1

In the control condition, the mean estimated amount of money that other in-group members kept for themselves was 377.03 JPY (SD = 144.14). The expected amount invested in the within- and between-group pools by other in-group members was 148.65 JPY (SD = 103.74) and 64.86 JPY (SD = 75.34), respectively. In the steal condition, the expected amount that other in-group members kept was 334.72 JPY (SD = 130.83). The estimated amount invested in the within- and between-group pools by in-group members was 163.89 JPY (SD = 93.05) and 91.67 JPY (SD = 84.09), respectively.

Regarding inferences on out-group members’ behavior, participants in the control condition expected that out-group members kept 360.81 JPY (SD = 159.05), invested 155.41 JPY (SD = 133.74) in the within-group pool, and invested 89.19 JPY (SD = 106.81) in the between-group pool. In the steal condition, the estimated amount that out-group members kept was 331.94 JPY (SD = 136.88). They expected out-group members to invest 195.83 JPY (SD = 99.55) and 83.33 JPY (SD = 75.59) in the within- and between-group pools, respectively.

S4. Means and Standard Deviations of Inference about others’ behavior in Study 2

In the control condition, the in-group members kept a mean estimated amount of 337.04 JPY (SD = 158.17). Participants expected other in-group members to invest 152.78 JPY (SD = 115.09) in the within-group pool and 125.00 JPY (SD = 128.40) in the between-group pool. In the steal condition, in-group members expected their peers to keep 279.59 JPY (SD = 153.07) for themselves and estimated the latter’s investment of 218.37 JPY (SD = 134.90) in the within-group pool and 110.20 JPY (SD = 80.35) in the between-group pool.

In the control condition, the out-group members estimated their peers to have kept 347.22 JPY (SD = 150.29) and invested 155.56 JPY (SD = 116.01) in the within-group pool and 120.37 JPY (SD = 126.09) in the between-group pool. In the steal condition, they expected out-group members to have kept 280.61 JPY (SD = 157.39) for themselves and invested 230.61 JPY (SD = 139.87) and 127.55 JPY (SD = 84.20) in the within- and between-group pools, respectively.

S5. Combined Analysis for the Behavioral Data

Given that Studies 1 and 2 employed identical procedures and study materials, the author combined the data from both experiments to increase the statistical power. The total sample size was 176. The number of participants in the control condition was 91, and that in the steal condition was 85. They, on average, kept 371.02 JPY (SD = 198.74) for themselves and invested 152.84 JPY (SD = 170.27) and 76.14 JPY (SD = 134.05) in the within- and between-group pools, respectively (see Figure S1).

A 2 (framing: control vs. steal) × 2 (pool: within- vs. between-group) mixed ANOVA on the amount of investment revealed a significant main effect of pool, *F*(1, 174) = 19.66, *p* <.001, *η*_p_^2^ = 0.10. In contrast, the main effects of framing (*F*(1, 174) = 2.41, *p* = .122, *η*_p_^2^ = 0.01) and the interaction effect (*F*(1, 174) = 2.59, *p* = .109, *η*_p_^2^ = 0.02) were not significant.

The mean amount of money invested in the between-group pool was 78.57 JPY (SD = 138.87) in the control condition and 73.53 JPY (SD = 129.47) in the steal condition, with no significant difference between them, *t*(174) = 0.25, *p* = .804, d = 0.04. In the steal condition, participants invested significantly less in the between-group pool than in the within-group pool (M = 179.41, SD = 176.49, *t*(84) = 4.19, *p* <.001, d = 0.69). Therefore, hypotheses S and W were not supported.

Using G*Power 3.1 (Faul, Erdfelder, Buchner, & Lang, 2009), sensitivity analysis of two tails t-test (power as 0.8) for hypothesis S revealed that the detectable effect size d was 0.425 and that for hypothesis W was 0.307.

Figure S1. Mean keep or invest amounts of each condition in the combined analysis (error bars represent standard errors).

S6. Combined Analysis with Data Exclusion for the Behavioral Data

Participants, on average, kept 366.67 JPY (SD = 198.73) for themselves and invested 154.24 JPY (SD = 169.86) and 79.09 JPY (SD = 137.61) in the within-group and between-group pools, respectively.

A 2 (framing: control vs. steal) × 2 (pool: within- vs. between-group) mixed ANOVA on the amount of investment revealed a significant main effect of pool, *F*(1, 163) = 16.37, *p* < .001, *η*_p_^2^ = 0.10. In contrast, the main effects of framing (*F*(1, 163) = 1.71, *p* = .192, *η*_p_^2^ = 0.01) and the interaction effect (*F*(1, 163) = 2.99, *p* = .086, *η*_p_^2^ = 0.02) were not significant.

The mean amount of money invested in the between-group pool was 85.00 JPY (SD = 146.35) in the control condition and 73.53 JPY (SD = 129.47) in the steal condition, with no significant difference between them, *t*(163) = 0.53, *p* = .594, d = 0.04. In the steal condition, participants invested significantly less in the between-group pool than in the within-group pool (M = 179.41, SD = 176.49, *t*(84) = 4.19, *p* < .001, d = 0.69). Therefore, hypotheses S and W were not supported.

S7. Combined Analysis for the Inference about others’ behavior

In the control condition, the mean estimated amount of money that other in-group members kept was 353.29 JPY (SD = 153.08), the amount invested in the within-group pool was 151.10 JPY (SD = 110.04), and the amount invested in the between-group pool was 100.55 JPY (SD = 113.41). In the steal condition, the mean estimated amount kept was 302.94 JPY (SD = 145.85), the amount invested in the within-group pool was 195.29 JPY (SD = 121.41), and the amount invested in the between-group pool was 102.35 JPY (SD = 81.98). A 2 (framing: control vs. steal) x 2 (pool: within- vs. between-group) mixed ANOVA on the estimated amount of money invested by in-group members revealed that the main effect of framing (*F*(1, 174) = 4.26, *p* = .041, *η*_p_^2^ = .02) and pool (*F*(1, 174) = 36.59, *p* <.001, *η*_p_^2^ = .17) were significant. The interaction effect was not significant (*F*(1, 174) = 3.19, *p* = .076, *η*_p_^2^ = .02).

Figure S2. Mean estimated keep or invest amounts by other in-group members of each condition in combined analysis (error bars represent standard errors).

In the control condition, the mean estimated amount of money that out-group members kept was 352.75 JPY (SD = 153.18), and that invested in the within- and between-group pools was 155.49 JPY (SD = 122.80) and 107.69 JPY (SD = 119.01), respectively. In the steal condition, the amount of money they expected out-group members to keep was 302.35 JPY (SD = 150.38), and that invested in the within- and between-group pools was 215.88 JPY (SD = 124.93) and 108.82 JPY (SD = 83.16). A 2 (framing: control vs. steal) × 2 (pool: within- vs. between-group) mixed ANOVA on the estimated amount of money invested by out-group members revealed that the main effect of framing (*F*(1, 174) = 7.22, *p* = .008, *η*_p_^2^ = .04) and pool (*F*(1, 174) = 36.39, *p* <.001, *η*_p_^2^ = .17) and their interaction effect (*F*(1, 174) = 5.33, *p* = .02, *η*_p_^2^ = .03) were significant. Multiple comparisons using the Holm method showed that the difference between the two framing conditions was not significant in the between-group pool (*p* = .95), but participants in the steal condition expected out-group members to invest more in the within-group pool, compared with the control condition (*p* = .001).

Figure S3. Mean estimated keep or invest amounts by out-group members of each condition in combined analysis (error bars represent standard errors).

S8. Auxiliary Variables: Identification and Social Dominance Orientation

The study measured identification with the in-group with the following four items: “Do you feel a strong connection with other members of your group?”, “To what extent do you feel integrated with other members of your group?”, “To what extent do you feel natural being with other members of your group?”, and “How important are the members of your group to you?” They were asked to respond to these four items on a nine-point scale from 1 = *not at all* to 9 = *very strongly*.

For Social Dominance Orientation (SDO), the study used the Japanese version of the SDO_6_ scale (Mifune and Yokota, 2018). Items include “In getting what your group wants, it is sometimes necessary to use force against other groups” and “Group equality should be our ideal”). The participants responded to these items on a seven-point scale from 1 = *strongly disagree/disapprove* to 7 = *strongly agree/favor*.

S9. Exploratory Analyses about Individual Differences

The study used the mean of the responses to the four items measuring in-group and out-group identification (α = .84) as the in-group and out-group identification score (α = .83). The relative in-group identification score was calculated by subtracting the out-group identification score from the in-group identification score. The mean of the 18 SDO items (α = .86) was used as the SDO score. Table S1 shows the correlations between these four scores and the amount of money kept or invested in the IPD–MD. No significant correlations were observed.

Table S1. Correlations between individual difference variables and behaviors in IPD–MD

|  | Total (N = 176) | | | | Control (N = 91) | | | | Steal (N = 85) | | | |
| --- | --- | --- | --- | --- | --- | --- | --- | --- | --- | --- | --- | --- |
|  | IgID | OgID | RID | SDO | IgID | OgID | RID | SDO | IgID | OgID | RID | SDO |
| Keep | -.09 | -.07 | -.04 | .05 | -.04 | -.04 | -.01 | .04 | -.12 | -.10 | -.04 | .08 |
|  | .26 | .37 | .60 | .52 | .71 | .71 | .90 | .73 | .26 | .36 | .72 | .44 |
| Within | .04 | -.03 | .09 | -.10 | .03 | -.03 | .10 | -.16 | .04 | -.02 | .07 | -.07 |
|  | .57 | .68 | .21 | .19 | .75 | .75 | .37 | .13 | .74 | .83 | .53 | .54 |
| Between | .07 | .14 | -.06 | .05 | .02 | .09 | -.09 | .14 | .14 | .19 | -.03 | -.04 |
|  | .35 | .06 | .42 | .48 | .88 | .38 | .39 | .19 | .19 | .08 | .78 | .70 |
| IgID: in-group identification, OgID: out-group identification, RID: relative in-group identification (IgID - OgID), SDO: social dominance orientation | | | | | | | | | | | | |
| The upper row shows correlation coefficients, and the lower row shows p-values (non-adjusted). | | | | | | | | | | | | |

References

Faul, F., Erdfelder, E., Buchner, A., & Lang, A.-G. (2009). Statistical power analyses using G*Power 3.1: Tests for correlation and regression analyses. Behavior Research Methods, 41, 1149-1160.

Mifune, N., & Yokota, K. (2018). The external validity of the relationship between social dominance orientation and political or discriminatory attitudes toward foreigners using a Japanese sample. *Japanese Journal of Social Psychology*, *34*(2), 94–101.
